# The immature dentate gyrus represents a shared phenotype of mouse models of epilepsy and psychiatric disease

**DOI:** 10.1111/bdi.12064

**Published:** 2013-04-06

**Authors:** Rick Shin, Katsunori Kobayashi, Hideo Hagihara, Jeffrey H Kogan, Shinichi Miyake, Katsunori Tajinda, Noah M Walton, Adam K Gross, Carrie L Heusner, Qian Chen, Kouichi Tamura, Tsuyoshi Miyakawa, Mitsuyuki Matsumoto

**Affiliations:** a CNS, Astellas Research Institute of America LLCSkokie, IL, USA; b Department of Pharmacology, Graduate School of Medicine, Nippon Medical SchoolTokyo, Japan; c Japan Science and Technology Agency, Core Research for Evolutional Science and TechnologySaitama, Japan; d Institute for Comprehensive Medical Science, Fujita Health UniversityAichi, Japan

**Keywords:** bipolar disorder, calbindin, calcium/calmodulin-dependent protein kinase II alpha, calretinin, dentate gyrus, doublecortin, neuronal immaturity, pilocarpine, schizophrenia, seizures

## Abstract

**Objectives:**

There is accumulating evidence to suggest psychiatric disorders, such as bipolar disorder and schizophrenia, share common etiologies, pathophysiologies, genetics, and drug responses with many of the epilepsies. Here, we explored overlaps in cellular/molecular, electrophysiological, and behavioral phenotypes between putative mouse models of bipolar disorder/schizophrenia and epilepsy. We tested the hypothesis that an immature dentate gyrus (iDG), whose association with psychosis in patients has recently been reported, represents a common phenotype of both diseases.

**Methods:**

Behaviors of calcium/calmodulin-dependent protein kinase II alpha (α-CaMKII) heterozygous knock-out (KO) mice, which are a representative bipolar disorder/schizophrenia model displaying iDG, and pilocarpine-treated mice, which are a representative epilepsy model, were tested followed by quantitative polymerase chain reaction (qPCR)/immunohistochemistry for mRNA/protein expression associated with an iDG phenotype. *In vitro* electrophysiology of dentate gyrus granule cells (DG GCs) was examined in pilocarpine-treated epileptic mice.

**Results:**

The two disease models demonstrated similar behavioral deficits, such as hyperactivity, poor working memory performance, and social withdrawal. Significant reductions in mRNA expression and immunoreactivity of the mature neuronal marker calbindin and concomitant increases in mRNA expression and immunoreactivity of the immature neuronal marker calretinin represent iDG signatures that are present in both mice models. Electrophysiologically, we have confirmed that DG GCs from pilocarpine-treated mice represent an immature state. A significant decrease in hippocampal α-CaMKII protein levels was also found in both models.

**Conclusions:**

Our data have shown iDG signatures from mouse models of both bipolar disorder/schizophrenia and epilepsy. The evidence suggests that the iDG may, in part, be responsible for the abnormal behavioral phenotype, and that the underlying pathophysiologies in epilepsy and bipolar disorder/schizophrenia are strikingly similar.

Incidence rates of epileptic patients who develop psychiatric symptoms have been reported to be significantly higher compared to other chronic conditions or healthy controls 1, 2. A recent study demonstrated that individuals with epilepsy have a 5.5-fold increased risk for developing broad psychosis, an almost 8.5-fold increased risk of having schizophrenia, and a 6.3-fold increased risk of having bipolar disorder 3. Clinically, epileptic patients can develop postictal psychosis or interictal depressive symptoms 4, 5 and often exhibit pronounced negative symptoms such as emotional withdrawal and blunted affect 6. Recent genetic studies have identified chromosome copy number variations, such as the 15q13.3 microdeletion, as conferring an increased risk for developing psychosis and epilepsy 7–9. Based on these and other findings, which suggest a genetic basis may underlie common phenotypes in epilepsy and psychosis, we hypothesize that an *immature dentate gyrus (iDG)* may represent a shared pathophysiology between the two diseases.

The hippocampal dentate gyrus is one of two brain regions where newborn neurons are generated throughout adulthood 10–12. During an approximately eight-week period, neural stem cells (NSCs) can differentiate into mature neurons or glial cells, and become incorporated into the hippocampal neural circuitry 13, 14. The normal developmental process from NSCs to mature granule cells [principal cells of the dentate gyrus (DG)] can be identified at various stages using specific neuronal markers, such as doublecortin for proliferative/progenitor cells, calretinin for immature neurons, and calbindin for mature neurons. The iDG phenotype represents a failure in the maturation of DG neurons 15 or a reversal of the state of matured neurons 16. Several mutant mice models of psychosis, where bipolar disorder/schizophrenia susceptibility genes have been manipulated, exhibit behavioral deficits related to psychosis and similar iDG phenotypes. These include mice with calcineurin conditional knock-out 17, 18, synaptosomal-associated protein of 25 kDa knock-in 18, schnurri-2 knock-out 18, and alpha-isoform of calcium/calmodulin-dependent protein kinase II heterozygous knock-out (α-CaMKII hKO) 15. Importantly, in a quantitative immunohistochemical analysis of postmortem human DGs, we found significantly increased calretinin immunoreactivity in bipolar disorder and schizophrenia patients with concomitantly decreased calbindin immunoreactivity in bipolar disorder patients 19. There was a strong relationship between elevations of calretinin and psychosis in human patients, which provided clinical evidence that the iDG may be an important phenotype of bipolar disorder/schizophrenia patients. Similarly, microarray analysis of schizophrenia postmortem brain tissue also demonstrated significant reductions in hippocampal calbindin gene expression 20.

The α-CaMKII hKO mice are the best characterized iDG mouse model with behavioral abnormalities such as hyperactivity, working memory deficits and social withdrawal, which are analogous to those in patients with bipolar disorder/schizophrenia 15, 19. Xing et al. 21 demonstrated that prefrontal cortical α-CaMKII mRNA expression in bipolar disorder patients was reduced to half of controls. Furthermore, we found that chronic lamotrigine treatment reversed social withdrawal and nest building deficits (commonly interpreted as a negative feature in an animal model) with a trend toward ameliorating poor working memory performance, while partially restoring the iDG of α-CaMKII hKO mice 22. Taken together, these data suggest that the α-CaMKII hKO mice have construct, face, and predictive validity as a model of bipolar disorder/schizophrenia, and that a reduction in α-CaMKII expression could be an important contributor to the iDG phenotype.

The pilocarpine-induced seizure is a well-established rodent model of temporal lobe epilepsy 22, 23–25. Based on the previous evidence that there is a permanent reduction of calbindin mRNA and protein expression from the hippocampus of rats following pilocarpine treatment 26, and a similar reduction in human postmortem epileptic brains 27, 28, we anticipated that the pilocarpine-induced seizure mice may also exhibit an iDG phenotype linking the pathophysiology of the two mouse models.

Our present study was based on the hypothesis that an iDG phenotype may be a core feature of both bipolar disorder/schizophrenia and epilepsy, and that the iDG may, in part, be responsible for the behavioral abnormalities that are common to both diseases. The implications of our study suggest that reversal of the phenotype may be beneficial for epileptic and bipolar disorder and/or schizophrenia patients.

## Materials and methods

### Animals

All procedures were approved by our Institutional Animal Care and Use Committee (Astellas Research Institute of America LLC, Skokie, IL, USA) and the Animal Care and Use Committee of Nippon Medical School (Tokyo, Japan). A total of 40 DBA/2J male mice (19 for vehicle and 21 for pilocarpine; Jackson Labs, Bar Harbor, ME, USA) were used in this study. DBA/2J mice were chosen because of the high mortality rates (90–95%) that occurred in C57BL/6 mice, even with the lowest effective pilocarpine dose for triggering seizures. We acknowledge that there are variations in seizure sensitivity between different mouse strains, and this could ultimately impact the iDG phenotype. However, the two mouse strains used in the present experiment, C57BL/6 and DBA/2J, yielded similar behavioral, immunohistochemical and molecular phenotypes, which suggest that the iDG phenomenon is represented across strains. α-CaMKII hKO mice, licensed from Massachusetts Institute of Technology, were backcrossed on a C57BL/6 background for more than 15 generations and maintained at Hilltop Labs (Scottdale, PA, USA). Mice were maintained on a 12-hour on, 12-hour off light cycle. Upon arrival at our facility, 40 DBA/2J, 17 wild-type (WT), and 17 α-CaMKII hKO male mice were singly housed to prevent excessive fighting. Although social isolation may negatively impact behaviors and potentially neurogenesis, we conducted pilot experiments and found housing conditions did not affect the iDG phenotype (data not shown). Food and water were available *ad libitum* except during seizure induction and behavioral testing. Pilocarpine-treated mice exhibited multiple daily spontaneous recurrent tonic-clonic seizures (SRSs) over a six-week period (eight weeks after pilocarpine) before behavioral testing. Animals were not previously exposed to the behavioral tasks prior to testing.

### Induction and characterization of status epilepticus (SE) in DBA/2J mice

**Fig. 1:**
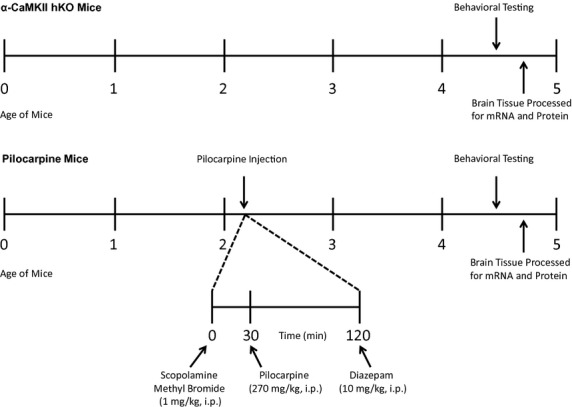
Time line of pilocarpine administration, behavioral testing, and tissue processing in DBA/2J and calcium/calmodulin-dependent protein kinase II heterozygous knock-out (α-CaMKII hKO) mice.

The induction of SE in DBA/2J mice is described in Figure 1. The optimal pilocarpine dose of 270 mg/kg for eliciting SE with minimal mortality rates was chosen based on previous studies 29–32. SE was achieved when the animal reached a state of continuous tremors, which is usually preceded by several periodic stage-5 (Racine scale) convulsive seizures 33. Control animals received identical drug treatment, except for intraperitoneal (i.p.) administration of 0.9% sodium chloride instead of pilocarpine.

### Spontaneous recurrent seizures and behavioral assessments

In this study, all of the mice injected with 270 mg/kg pilocarpine experienced SE. Because DBA/2J mice have been shown to exhibit very low mortality rates following administration of various concentrations of pilocarpine 29, we felt they were a suitable model of chronic epilepsy despite the different genetic background from the α-CaMKII hKO mice. The monitoring and confirmation of SRS commenced three weeks following pilocarpine induction by daily videotaping (10 a.m. to 4 p.m.) each mouse for two consecutive days. All pilocarpine-treated mice exhibited on average 1–3 SRSs with stage-5 severity 33 during the 6-hour recording period, and thus were considered *epileptic*. All experiments were performed between 8 a.m. and 5 p.m. For all behavioral tests, except for social investigation, the apparatus was wiped thoroughly with Anlage QTB disinfectant and deodorizer (Quiplabs, Wilmington, DE, USA) after removal of each mouse.

### Drugs

Pilocarpine hydrochloride, scopolamine methyl bromide (Sigma-Aldrich, St. Louis, MO, USA), and diazepam (Hospira, Inc., Lake Forest, IL, USA) were freshly dissolved in sterile 0.9% NaCl prior to i.p. administration.

### Behavioral measurements

#### Open-field test

Using the Ethovision XT v.7 software program (Noldus Information Technology, Leesburg, VA, USA) to track movement in infrared light, total horizontal distance traveled over a 30-min period in a 50 × 50 × 50 cm (L × W × H) transparent plexiglass chamber (Med Associates, St. Albans, VT, USA) was measured.

#### Working memory (Y-maze)

Working memory deficits during a spontaneous delayed alternation task were assessed using an automated Y-maze (Med Associates) with three electronically controlled gray guillotine doors and infrared detection beams. The task began when the animal was randomly placed in any 37-cm arm with all doors closed for 30 sec, after which it was free to choose to enter any arm (*start arm*). One minute later, the animal could choose to enter any arm (*choice arm*). Once a choice had been made, all doors were closed for 30 sec, after which it could choose any arm (*final arm*). There were five trials in total with an intertrial interval of 1 min. Maximum time allowed to complete this task was 15 min. A perfect score was attained when the animal did not re-enter an arm previously chosen within a particular trial. Percent correct was calculated by averaging all five trials. All arms of the maze were unbaited.

#### Social investigation

Ovariectomized Balb/c female mice (Hilltop Labs) at four months of age were used to examine social investigative behaviors in the male's home cage (20 × 30 × 13 cm) for a duration of 5 min. Investigation times were counted when the male's head was in contact with the female. Trials were videotaped and were manually scored with investigators blind to genotype and treatment.

#### Sensitivity of α-CaMKII hKO mice to pilocarpine

Mice (n = 5/dose) were administered a single injection of pilocarpine (200, 230, 250, 300, or 350 mg/kg, i.p. for WT; 30, 50, 70, or 100 mg/kg, i.p. for mutants). The doses were determined from pilot studies and literature review. The lowest dose of pilocarpine required to elicit a seizure was recorded.

### Immunohistochemistry

A separate cohort of α-CaMKII hKO (n = 2) and pilocarpine-treated mice with SRS (n = 2) were used for qualitative immunohistochemistry. Animals were perfused intracardially with 0.9% NaCl followed by 4% ice-cold paraformaldehyde. The brains were post-fixed overnight then transferred to a 30% sucrose solution in 0.1 M phosphate-buffered saline (PBS), pH 7.2, for a minimum of three days before sectioning. Coronal sections of the hippocampus were cut on a cryostat at 20 μm. Sections were then washed three times for 10 min each with 0.3% TX-100 in PBS followed by blocking at room temperature with 2.5% normal donkey serum (Jackson Immunolabs, West Grove, PA, USA) in 0.3% TX-100 in PBS. Primary antibodies [calbindin: 1:3000, Swant; calretinin: 1:3000, Swant; doublecortin (DCX): 1:500] (Cell Signaling Technology, Inc., Danvers, MA, USA) were incubated overnight at 4°C. Sections were then subsequently rinsed three times for 10 min each with 0.3% TX-100 in PBS before incubation of secondary antibodies for 40 min at room temperature [Cy2/3 donkey anti-rabbit (1:800); Jackson Immunolabs]. Finally, sections were washed as described above and mounted on gelatinized glass slides and coverslipped with Fluoromount-G mounting medium (Southern Biotech, Birmingham, AL, USA). Time-matched exposures of DG images were taken at 20× with a Keyence BZ-9000 microscope (Keyence, Osaka, Japan).

### mRNA isolation, cDNA synthesis and quantitative polymerase chain reaction (qPCR)

Following behavioral testing, animals were decapitated and the brains were removed quickly and sliced coronally on a vibratome at 500-μm thickness in ice-cold PBS. Two slices of the dorsal hippocampus (−1.4 to −2.4 mm relative to bregma) were acquired. Using a standard 300-μl pipette tip (0.46 μm diameter), tissue from the DG was excised from both hemispheres. The punch included both dorsal and ventral blades of the granule cell layer toward the midline, including a portion of the hilus. A total of four pipette punches (two brain slices, both hemispheres) were homogenized in 350 μL of lysis buffer. Total RNA from the dentate gyrus was prepared by RNeasy Micro Kit (Qiagen, Germantown, MD, USA) with DNase I treatment. One hundred nanograms of total RNA was reverse-transcribed by random priming to synthesize single-strand cDNA (SuperScript III; Invitrogen, Grand Island, NY, USA). The qPCR reaction contained 15-times diluted cDNA from the synthesis reaction, 1× FastSYBR reagent (Applied Biosystems, Grand Island, NY, USA), and 200 nM of specific forward and reverse primers in a 10-μL volume of reaction. Mouse genomic DNA was used in the standard curve reaction for absolute quantification. qPCR reaction and measurement were carried out with the ViiA 7 Real-Time PCR System (Applied Biosystems). The PCR reaction conditions were 95°C, 20 sec and 40 cycles of 95°C, 1 sec and 60°C, 20 sec followed by a dissociation curve step to verify single amplicon in the reaction. The expression value of the test genes was normalized with the 18S rRNA signal and shown as relative expression. qPCR mouse primers are listed in Table 1.

**Table 1:** Mouse primer sequences for RT-qPCR

Calbindin	Forward: 5′-GCTCCGCGCACTCTCAA-3′
	Reverse: 5′-TGACTGCAGGTGGGATTCTG-3′
Calretinin	Forward: 5′-CGGAGCTGGCGCAGAT-3′
	Reverse: 5′-CTGCCTGAAGCACAAAAGGAA-3′
Doublecortin	Forward: 5′-TTCGTAGTTTTGAGCGTTGCT-3′
	Reverse: 5′-GAGGCAGGTTAATGTTGTCAG-3′
αCaMKII	Forward: 5′-TGGGTTTGGCTCTTGTATGGA-3′
	Reverse: 5′-AAGAAAACAGTGCAGACAGGAGATC-3′
Tdo2	Forward: 5′-GGCAGAGTTCCGGAAGCA-3′
	Reverse: 5′-CATGACGCTTCTCATCAAACAA-3′
Bdnf	Forward: 5′-GCCCTGCGGAGGCTAAGT-3′
	Reverse: 5′-GGATGGCCACTCAGAAATTCC-3′
Drd1a	Forward: 5′-ACAGCAGCCCCTCCGATAG-3′
	Reverse: 5′-GTTAGACCTGGGCAGATGAAG-3′
Grp	Forward: 5′-TGAATCCCCGTCCCTGTATG-3′
	Reverse: 5′-TACCCCCTCAGCTGCTCCTT-3′
Il1r1	Forward: 5′-AAACCTCTGCTTCTTGACAACGT-3′
	Reverse: 5′-AGCCACATTCCTCACCAACAG-3′
Dsp	Forward: 5′-GCTCCATTACCAAGACTTCATC-3′
	Reverse: 5′-TGTCGTCGTCTCCAAACATCT-3′
18S rRNA	Forward: 5′-GTAACCCGTTGAACCCCATT-3′
	Reverse: 5′-CCATCCAATCGGTAGTAGCG-3′

Mouse forward and reverse primers (Sigma-Aldrich) for real-time quantitative polymerase chain reaction (RT-qPCR).

αCaMKII = calcium/calmodulin-dependent protein kinase II alpha; 18S rRNA = 18S ribosomal RNA; Bdnf = brain-derived neurotrophic factor; Drd1a = dopamine receptor D1A; Dsp = desmoplakin; Grp = gastrin-releasing peptide; Il1r1 = interleukin 1 receptor, type 1; Tdo2 = tryptophan 2,3-dioxygenase.

### Western blot

From the remaining cohort of mice (n = 7, α-CaMKII hKO; n = 6, pilocarpine) whole hippocampus from one hemisphere was used for processing protein. Cells were lysed in a modified radioimmunoprecipitation assay buffer containing (in mM): 150 NaCl, 50 EDTA (pH 7.5), 50 sodium β-glycerophosphate, 50 NaF, 5 sodium pyrophosphate, 2 EDTA, 2 EGTA, 1 DTT, 1 phenylmethylsulfonyl fluoride, 1 sodium orthovanadate with 1% Triton X-100, 10 μg/mL leupeptin and 10 μg/mL aprotinin (Sigma-Aldrich). Equal amounts of lysates were resolved on a 4–12% sodium dodecyl sulfate (SDS) -polyacrylamide gel and transferred to a polyvinylidene fluoride membrane. The membrane was blocked in PBS containing 5% nonfat dry milk for 1 hour and then incubated with primary antibodies in PBS containing 5% nonfat milk overnight at 4°C. Primary antibodies used were: rabbit anti-CaMKIIα, 1:2000 (Abcam, Cambridge, MA, USA); mouse anti-beta actin, 1:2000 (Cell Signaling Technology, Inc., Danvers, MA, USA). Horseradish peroxidase-labeled secondary antibodies were applied in PBS containing 5% nonfat dry milk for 1 hour. Secondary antibodies: rabbit anti-immunoglobulin G (anti-IgG), 1:3000 (GE Healthcare, Pittsburg, PA, USA); mouse anti-IgG, 1:3000 (GE Healthcare). All samples were normalized to B-actin. Protein was visualized by using an enhanced chemiluminescence (ECL) detection system (Amersham, Pittsburg, PA, USA) on a ChemiDoc XRS Scanner (BioRad, Hercules, CA, USA).

### *In vitro* electrophysiology

Whole-cell recordings and field potential recordings were made using acute hippocampal slices. Electrophysiological experiments were performed 6–12 weeks after pilocarpine administration. Mice were decapitated under halothane anesthesia and both hippocampi were isolated. Transverse hippocampal slices (380 μm) were cut using a tissue slicer and maintained in a humidified interface holding chamber at room temperature until use. Electrophysiological recordings were made in a submersion-type chamber superfused at 2 mL/min with a standard saline cocktail that was comprised of (in mM): NaCl, 125; KCl, 2.5; NaH_2_PO_4_, 1.0; NaHCO_3_, 26.2; glucose, 11;CaCl_2_, 2.5; MgCl_2_, 1.3 (equilibrated with 95% O_2_/5% CO_2_) and maintained at 27–27.5°C. Blind whole-cell patch-clamp recordings and field potential recordings were made using a Multiclamp 700B amplifier (Molecular Devices, Sunnyvale, CA, USA) as described previously 15, 16. Whole-cell current-clamp recordings were made from dentate granule cells with a glass pipette filled with a solution composed of (in mM): potassium gluconate, 140; HEPES, 20; NaCl, 8; MgATP, 2; Na_2_GTP, 0.3; EGTA, 0.05 (pH adjusted to 7.2 with KOH). Hyperpolarizing and depolarizing currents (400 msec) were injected through the recording pipette to measure the input resistance and to assess properties of action potential firing, respectively. Field excitatory postsynaptic potentials (EPSPs) arising from mossy fiber (MF) synapses were recorded in the stratum lucidum with a glass electrode filled with 2 M NaCl. EPSPs were evoked by bipolar stimulating electrodes placed in the granule cell layer (GCL) at the frequency of 0.05 Hz unless otherwise specified. A criterion of 85% blockage of EPSPs was used by the group II metabotropic glutamate receptor agonist (2S,2′R,3′R)-2-(2′,3′-dicarboxycyclopropyl) glycine (DCG-IV; Tocris Bioscience, Bristol, UK) to identify the MF input.

### Data analysis

Data were analyzed in GraphPad Prism v5.03 (GraphPad Software, La Jolla, CA, USA) using a two-tailed *t*-test that compared the respective treatment groups. For the open field task, data were analyzed using a two-way ANOVA with *genotype* as the between-groups factor and *time* as the within-groups factor. Bonferroni post-tests for comparing individual means were used whenever appropriate.

## Results

### Behavioral measurements

#### Open field test

**Fig. 2:**
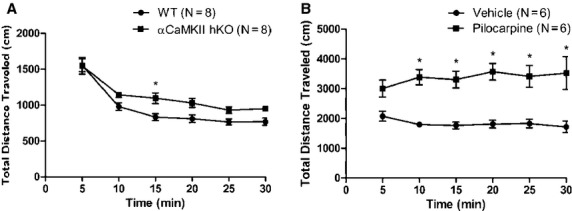
Hyperactivity in the open field. Average cumulative distance traveled ± SEM was assessed in calcium/calmodulin-dependent protein kinase II heterozygous knock-out (α-CaMKII hKO) **(A)** and pilocarpine-treated **(B)** mice compared to wild-type (WT) and vehicle-treated controls, respectively, over a 30-min period in darkness. *p < 0.05.

α-CaMKII hKO mice exhibited significantly higher levels of locomotion in the open field test compared with WT controls [*F*(1,70) = 5.33, p < 0.05], but both groups demonstrated habituation over a 30-min period [*F*(5,70) = 78.8, p < 0.0001]. Although the mice displayed identical locomotion times during the first 5 min, the mutants showed less habituation to the chamber for the remainder of the test compared to controls, resulting in a significant time × genotype interaction [*F*(5,70) = 2.41, p < 0.05] (Fig. 2A). Pilocarpine-treated mice showed a substantially significant increase in hyperactivity compared to WT controls [*F*(1,50) = 24.2, p < 0.001] with no habituation to the chamber (Fig. 2B). Their locomotor activity was much more pronounced than that of the α-CaMKII hKO mice [*F*(1,60) = 80.0, p < 0.0001].

#### Working memory (Y-maze)

**Fig. 3:**
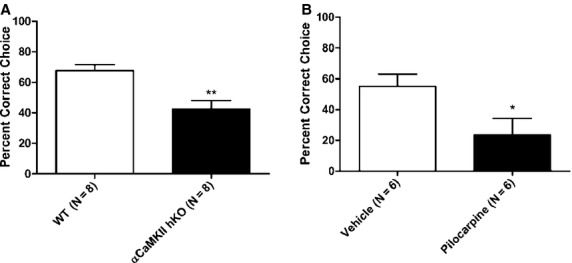
Working memory deficits in the Y-maze test. Average percent correct choice ± SEM over five trials was assessed in calcium/calmodulin-dependent protein kinase II heterozygous knock-out (α-CaMKII hKO) **(A)** and pilocarpine-treated **(B)** mice compared to wild-type (WT) and vehicle-treated controls, respectively. A correct trial was defined as non-repeat entry into a previously visited arm on a single trial. The test included a 30-sec delay between the closing of the guillotine doors following the choice (second) arm and the opening of the doors at the start of the final (third) arm of the maze. *p < 0.05, **p < 0.01.

A spontaneous delayed alternation task was used to assess working memory performance. α-CaMKII hKO mice showed significant impairment in the Y-maze compared to controls (*t* = 3.62, df = 14, p < 0.01; Fig. 3A), as did pilocarpine-treated mice at a 30-sec delay (*t* = 2.34, df = 10, p < 0.05; Fig. 3B). One observable key difference is that the majority of pilocarpine mice exhibited perseveration in this task by entering either the same arm repeatedly or alternating between two preferential arms.

#### Social interaction

**Fig. 4:**
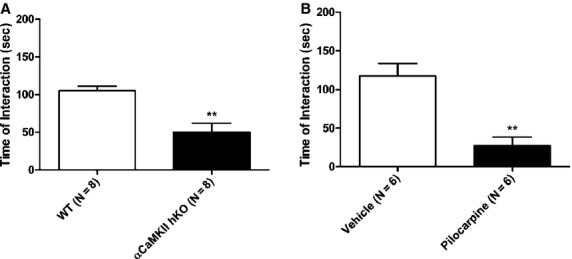
Decreased social investigation with the female. Average total investigation times ± SEM, including body and anogenital sniffing, by calcium/calmodulin-dependent protein kinase II heterozygous knock-out (α-CaMKII hKO) **(A)** and pilocarpine-treated **(B)** mice compared to wild-type (WT) and vehicle-treated controls, respectively, toward a Balb/c ovariectomized female mouse were recorded during a 5-min interval in the subject's home cage. **p < 0.01.

In this task, examining free interaction with a stimulus mouse, α-CaMKII hKO mice generally showed periods of investigatory behavior during the first 2 min of the 5-min test, but exhibited decreased interaction thereafter with only sporadic encounters. Pilocarpine-treated mice appeared socially aversive with little to no pursuant activity of the stimulus female. Despite their robust hyperactivity in the open field, they appeared hypoactive during this task. In either case, a similar and robust reduction in social interaction times was observed in the α-CaMKII hKO (*t* = 4.61, df = 10, p < 0.001; Fig. 4A) and pilocarpine-treated mice (*t* = 4.12, df = 14, p < 0.002) compared to WT controls (Fig. 4B).

### Psychiatric and epileptic deficits in pilocarpine-induced seizure and α-CaMKII hKO mice

**Fig. 5:**
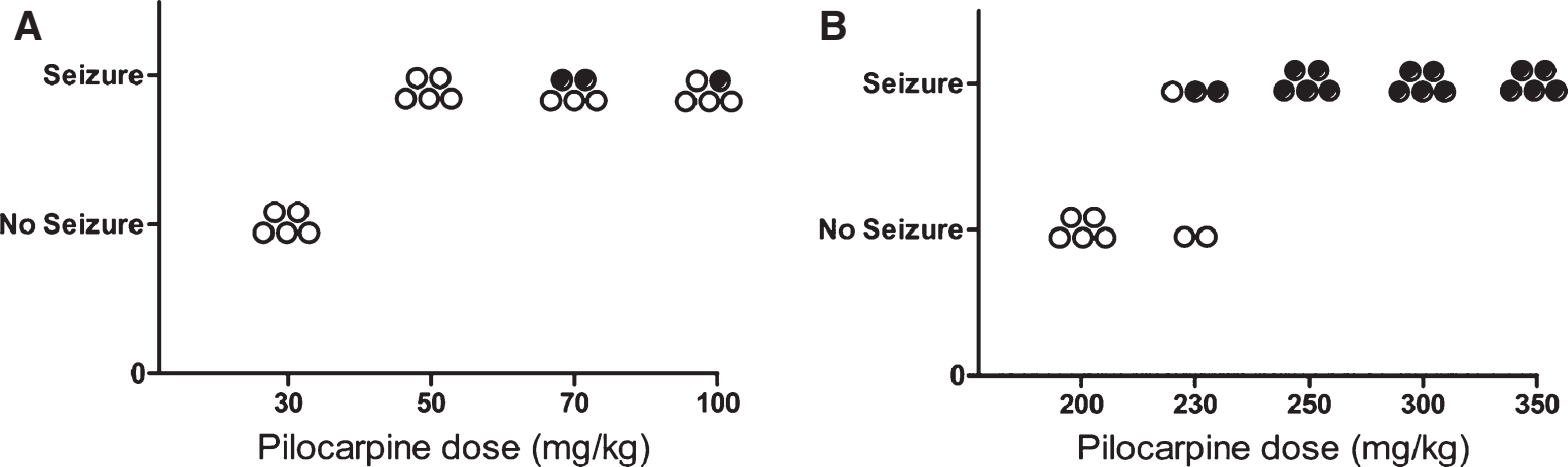
Heightened sensitivity to pilocarpine in calcium/calmodulin-dependent protein kinase II heterozygous knock-out (α-CaMKII hKO) mice. Various doses of pilocarpine were administered to α-CaMKII hKO (n = 5/dose) **(A)** and wild-type (n = 5/dose) **(B)** mice to assess seizure threshold. 50 and 230 mg/kg of pilocarpine were found to be the minimal dose required to elicit seizures in α-CaMKII hKO and wild-type mice, respectively. Open circles represent mice that survived the treatment; closed circles represent mice that died within 90 min of pilocarpine injection.

We have shown that pilocarpine-treated mice showed similar psychiatric-analogous features to α-CaMKII hKO mice, including hyperactivity, poor working memory performance, and social withdrawal. Conversely, we also examined whether α-CaMKII hKO mice exhibited characteristics of epilepsy. Although we observed that α-CaMKII hKO mice did not have SRS, they demonstrated an approximately 4× higher sensitivity (lower seizure thresholds) to pilocarpine compared to WT mice when tested at various dosages (Fig. 5A and B). It is surprising that despite their lower seizure thresholds, supra-threshold dosages (70 and 100 mg/kg) did not result in the death of all α-CaMKII hKO mice tested. It is possible that the pilocarpine challenge may have transiently enhanced compensatory inhibitory mechanisms in the mutant mice. Generally, we have observed that mortality occurs immediately following one of the many possible stage-5 seizures prior to the onset of SE. Higher dosages of pilocarpine were not tested in these mice because it was important to establish seizure threshold sensitivity rather than lethal dose. Surprisingly, mortality rates in WT mice were achieved at a pilocarpine dose of 250 mg/kg. Other pilocarpine studies employed dosages of 250–350 mg/kg, which we systemically tested.

### Expression of DG maturation markers in the epilepsy model and α-CaMKII hKO mice

**Fig. 6:**
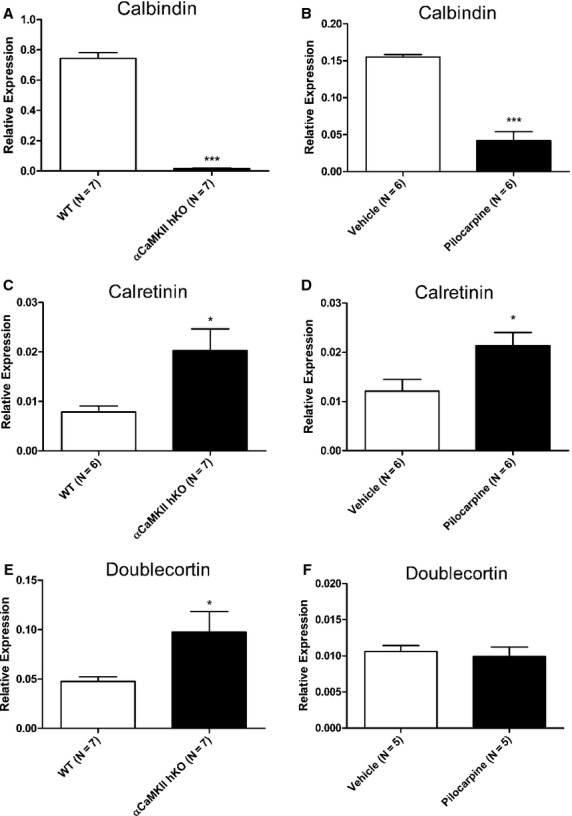
mRNA expression of dentate gyrus maturation markers. Average mRNA expression ± SEM of calbindin, calretinin, and doublecortin in calcium/calmodulin-dependent protein kinase II heterozygous knock-out (α-CaMKII hKO) **(A, C, E)** and pilocarpine-treated **(B, D, F)** mice, respectively, compared to controls. Average values were normalized to the housekeeping gene 18S rRNA. *p < 0.05, ***p < 0.001. WT = wild-type.

**Fig. 7:**
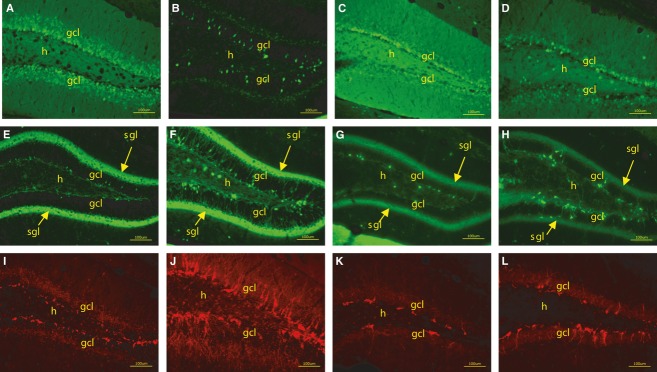
Representative immunofluorescence staining of maturation markers in the dentate gyrus (DG) at 20× magnification. A comparison was made between wild-type controls and calcium/calmodulin-dependent protein kinase II heterozygous knock-out (α-CaMKII hKO) DG sections stained with anti-calbindin **(A, B)**, calretinin **(E, F)**, and doublecortin **(I, J)**, respectively. An identical comparison was made between DG sections from vehicle- and pilocarpine-treated mice stained with anti-calbindin **(C, D)**, calretinin **(G, H)**, and doublecortin **(K, L)**, respectively. Scale bar = 100 μm. h = hilus; gcl = granule cell layer; sgl = supragranular layer.

Tissue from the DG of α-CaMKII hKO and pilocarpine-treated mice was examined for changes in gene expression to characterize the development of adult neurogenesis. Relative expression values for each gene were normalized to 18S ribosomal RNA. 18S values were not statistically different between WT/vehicle and α-CaMKII hKO/pilocarpine groups (WT: 51.4 ± 6.58; α-CaMKII hKO: 62.74 ± 11.80; DBA vehicle: 141.80 ± 9.34; pilocarpine: 143.64 ± 12.61 SEM). We measured the expression of doublecortin, a marker for progenitor cells, calretinin, a marker for immature neurons, and calbindin, a marker for mature neurons. The most dramatic change was seen in calbindin mRNA expression for α-CaMKII hKO mice, which was over 40-fold lower than for WT controls (*t* = 19, df = 12, p < 0.0001; Fig. 6A). In pilocarpine-treated mice, a 3.7-fold reduction was observed (*t* = 8.91, df = 10, p < 0.0001; Fig. 6B). As seen in Figure 7A–D, calbindin immunoreactivity of the granule cell layer was nearly absent in α-CaMKII hKO mice, and to a lesser extent in the pilocarpine-treated mice compared to controls. The mRNA expression of calretinin was significantly increased in α-CaMKII hKO (*t* = 2.52, df = 11, p < 0.05; Fig. 6C) and pilocarpine-treated mice (*t* = 2.59, df = 10, p < 0.05; Fig. 6D) compared to their controls. Calretinin immunoreactive cells appeared to localize near the subgranular zone, especially in WT or vehicle-treated controls. However, in α-CaMKII hKO and pilocarpine-treated mice, immunoreactive cells could also be seen in the hilus, which are presumably either mossy cells or spiny multipolar cells 34, as distinguished by their shape, and throughout the granule cell layer (Fig. 7E–H). Doublecortin mRNA expression was higher in the α-CaMKII hKO mice (*t* = 2.32, df = 12, p < 0.05; Fig. 6E), but did not change in pilocarpine-treated mice compared to controls when examined at two months after pilocarpine administration (Fig. 6F). Robust doublecortin immunoreactivity of cell bodies within the subgranular zone and its processes extending through the granule cell layer was observed in α-CaMKII hKO mice. Much more muted immunoreactive expression was seen in pilocarpine-treated mice (Fig. 7I–L). We did, however, observe substantial elevation of doublecortin immunoreactivity in pilocarpine-treated mice after one month (data not shown), which is consistent with previous reports 35, 36.

### Gene expression of iDG markers in the epilepsy model and α-CaMKII hKO mice

**Fig. 8:**
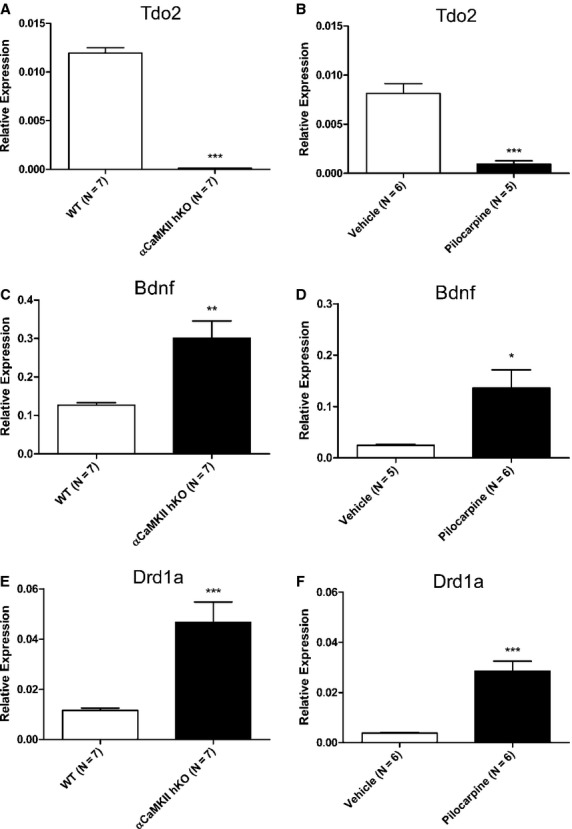
Tryptophan 2,3-dioxygenase (Tdo2), brain-derived neurotrophic factor (Bdnf), and dopamine 1 receptor (Drd1a) mRNA expression. Average mRNA expression ± SEM of Tdo2, Bdnf, and Drd1a genes from the dentate gyrus of calcium/calmodulin-dependent protein kinase II heterozygous knock-out (α-CaMKII hKO) **(A, C, E)** and pilocarpine-treated **(B, D, F)** mice, respectively, compared to controls. Average values were normalized to the housekeeping gene 18S rRNA. *p < 0.05, **p < 0.01, ***p < 0.001. WT = wild-type.

**Fig. 9:**
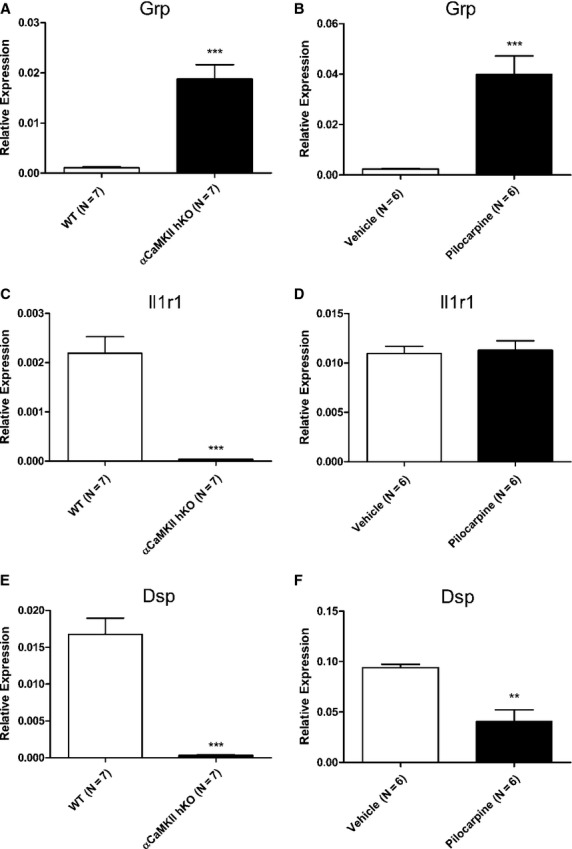
Gastrin-releasing peptide (Grp), interleukin-1 receptor (Il1r1), and desmoplakin (Dsp) mRNA expression. Average mRNA expression ± SEM of Grp, Il1r1, and Dsp genes from the dentate gyrus of calcium/calmodulin-dependent protein kinase II heterozygous knock-out (α-CaMKII hKO) **(A, C, E)** and pilocarpine-treated **(B, D, F)** mice, respectively, compared to controls. Average values were normalized to the housekeeping gene 18S rRNA. **p < 0.01, ***p < 0.001. WT = wild-type.

Tryptophan 2,3-dioxygenase (Tdo2), brain-derived neurotrophic factor (Bdnf), dopamine 1 receptor (Drd1a), gastrin-releasing peptide (Grp), interleukin-1 receptor (Il1r1), and desmoplakin (Dsp) are genes that were previously shown to be dysregulated in the DG of α-CaMKII hKO mice 15, 16. mRNA expression of Tdo2 in α-CaMKII hKO (*t* = 22.0, df = 12, p < 0.0001; Fig. 8A) and pilocarpine-treated mice (*t* = 6.15, df = 9, p < 0.001; Fig. 8B) was dramatically reduced compared to controls. Similar to the increase in mRNA expression of doublecortin and calretinin, α-CaMKII hKO mice had a significant increase in mRNA expression of the neurotrophic factor Bdnf compared to WT controls (*t* = 3.89, df = 12, p < 0.01) (Fig. 8C). The same increase in Bdnf mRNA expression (*t* = 2.87, df = 9, p < 0.05) was evident in pilocarpine-treated mice (Fig. 8D). We additionally observed abnormally high mRNA expression levels of Drd1a from the DG of α-CaMKII hKO (*t* = 4.36, df = 12, p = 0.001; Fig. 8E) and pilocarpine-treated mice (*t* = 6.12, df = 10, p < 0.0001; Fig. 8F), which is consistent with enhanced D1-like receptor-mediated synaptic modulation at the mossy fiber synapse. Grp mRNA was shown to be highly elevated in the DG of α-CaMKII hKO mice (*t* = 6.12, df = 12, p < 0.0001) and pilocarpine-treated mice (*t* = 5.19, df = 10, p < 0.001) compared to controls (Fig. 9A and B). Two more genes whose mRNA expression was dysregulated in α-CaMKII hKO mice compared to WT mice were Il1r1 and Dsp (*t* = 6.40, df = 12, p < .0001, Il1r1; and *t* = 7.65, df = 12, p < .0001, Dsp; Fig. 9C and E). While Il1r1 mRNA expression was not changed, Dsp mRNA expression was significantly decreased in pilocarpine-treated mice relative to controls (n.s., Il1r1; and *t* = 4.47, df = 10, p < 0.01, Dsp; Fig. 9D and F).

### Electrophysiological properties of DG granular cells in the epilepsy model

**Fig. 10:**
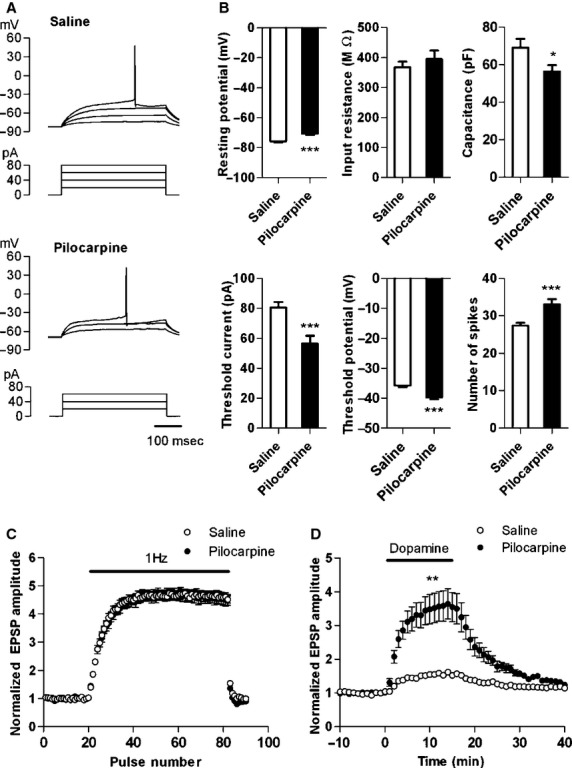
Electrophysiological properties of dentate granule cells and mossy fiber synapses in pilocarpine-treated mice. **(A)** Sample recordings of granule cell action potentials evoked by depolarizing currents. **(B)** Pooled data showing somatic electrophysiological properties of granule cells in saline-treated (n = 23) and pilocarpine-treated mice (n = 22). The resting membrane potential, input resistance, membrane capacitance calculated from input resistance and membrane time constant, threshold current intensity to evoke a single spike, spike threshold potential, and maximal number of spikes during sustained depolarization are shown. **(C)** Frequency facilitation induced by 1-Hz stimulation is intact at the mossy fiber-CA3 synapse (n = 8 each). **(D)** Dopamine-induced synaptic potentiation mediated by D1-like receptors is enhanced in pilocarpine-treated mice (saline: n = 5, pilocarpine: n = 6). Dopamine (10 μM) was applied in bath as indicated. *p < 0.05, **p < 0.01, ***p < 0.001.

We made whole-cell current clamp recordings from dentate granule cells (GCs) to examine the physiological properties of these cells. Pilocarpine-treated GCs had depolarized resting membrane potentials (−70.8 ± 0.8 pilocarpine versus −75.8 ± 0.6 mV vehicle; *t* = 5.023, df = 43, p < 0.0001; Fig. 9B, top left), intact input resistance (395.0 ± 28.4 pilocarpine versus 368.3 ± 18.1 MΩ vehicle) (*t* = 0.7931, df = 35, p = 0.433; Fig. 10B, top center), and smaller membrane capacitance (56.3 ± 3.4 pilocarpine versus 69.1 ± 4.7 pF vehicle; *t* = 2.180, df = 43, p < 0.05) compared to GCs from vehicle-treated mice (Fig. 10B, top right). The current intensity to evoke an action potential was smaller (56.4 ± 5.4 pilocarpine versus 80.4 ± 3.7 pA vehicle; *t* = 3.705, df = 43, p < 0.001; Fig 9B, bottom left), the spike threshold potential was lower (−39.7 ± 0.7 pilocarpine versus −35.8 ± 0.5 mV vehicle; *t* = 4.417, df = 43, p < 0.0001; Fig. 10B, bottom center), and more spikes were observed during sustained depolarization (33.1 ± 1.4 pilocarpine versus 27.4 ± 0.8 mV vehicle; *t* = 3.647, df = 33, p < 0.001; Fig. 10B, bottom right), indicating higher excitability of GCs from pilocarpine-treated compared to vehicle-treated mice. We also examined synaptic transmission between the mossy fiber axons of GCs and CA3 pyramidal cells. The prominent frequency facilitation characteristic of this synapse was intact 37, 38 (358.5 ± 21.2 pilocarpine versus 363.0 ± 13.4% increase vehicle; *t* = 0.1792, df = 14, p = 0.860; Fig. 10C), while a dopamine-induced synaptic potentiation mediated by D1-like receptors was greatly enhanced in the GCs from pilocarpine-treated mice (255.9 ± 48.3 pilocarpine versus 56.6 ± 7.6% increase vehicle; *t* = 4.080, df = 5, p < 0.01; Fig. 10D). Overall, GCs from pilocarpine-treated mice share some of the electrophysiological properties with GCs from α-CaMKII hKO mice 15. Furthermore, GCs from both mice share properties, especially regarding heightened excitability, observed in GCs from chronic fluoxetine-treated mice that exhibit a ‘dematuration’ of GCs 16, 38.

### α-CaMKII mRNA and protein in the epilepsy model and α-CaMKII hKO mice

**Fig. 11:**
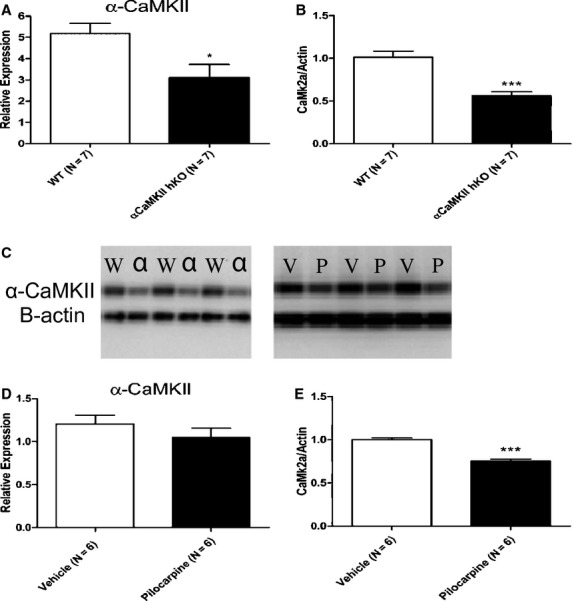
Calcium/calmodulin-dependent protein kinase II (α-CaMKII) mRNA and total protein expression. Average mRNA expression ± SEM of α-CaMKII from α-CaMKII heterozygous knock-out (hKO) **(A)** and pilocarpine-treated **(D)** mice. Bar graphs show significant reductions of average total hippocampal α-CaMKII protein expression ± SEM in α-CaMKII hKO **(B)** and pilocarpine-treated **(E)** mice, with western blot **(C)** normalized to B-actin. W = wild-type; α = α-CaMKII hKO; V = vehicle; P = pilocarpine. Average mRNA values were normalized to the housekeeping gene 18S rRNA. *p < 0.05, ***p < 0.001.

We confirmed by qPCR and western blots that the levels of α-CaMKII mRNA in the DG (*t* = 2.69, df = 12, p < 0.05) and total protein in the whole hippocampus (*t* = 5.33, df = 12, p < 0.001) of the α-CaMKII mice were approximately half those of WT controls (Fig. 11A–C). α-CaMKII mRNA from pilocarpine-treated mice did not differ from that of vehicle controls (Fig. 11D), but total hippocampal protein was significantly reduced by approximately 25% of controls (*t* = 7.27, df = 10, p < 0.0001; Fig. 11C and E). B-actin levels were quantified based on measured intensity/mm^2^ and were not statistically different between treatment groups (WT: 503254 ± 28431; α-CaMKII hKO: 479428 ± 35037; DBA vehicle: 971213 ± 24901; pilocarpine: 956935 ± 22892).

## Discussion

The present study provides evidence that α-CaMKII hKO and pilocarpine-treated seizure mice share many common phenotypes, including behavioral changes, some GC electrophysiological properties, neuronal maturation marker changes and iDG-related genes, that were previously reported to be dysregulated in several mouse models of psychiatric disease 15, 16. Recently, we demonstrated that postmortem samples from psychotic patients had an iDG feature 19. The implications of our data are that the iDG is a common feature of both psychosis and epilepsy.

### Neuronal maturation marker expression in the immature dentate gyrus

An important finding in our study was the significant reduction in mRNA and immunoreactivity of the mature neuronal marker calbindin in the dorsal DG in both α-CaMKII hKO and pilocarpine-treated mice. This result, along with an over-expression of the immature neuronal marker calretinin, suggests that there is an elevation of immature GCs and a reduction of mature GCs in both mouse models 15.

Although calbindin has been frequently used as a mature neuronal marker, it also functions as an interneuron marker and calcium-binding protein that can buffer excess intracellular calcium. We observed the robust decrease in calbindin immunoreactivity to be presumably localized in GCs, which are primarily principal cells within the GC layer. Functionally, a decrease in calbindin immunoreactivity may represent a shift in seizure threshold favoring increased excitation and/or decreased inhibition, which can ultimately influence cellular excitability 40, 41. Given that the α-CaMKII hKO mice exhibited more than a 4-fold increase in sensitivity to pilocarpine compared to vehicle controls, and demonstrated a substantial decrease in mRNA calbindin expression (similarly to the pilocarpine-treated mice), it is, perhaps, not surprising that intracellular calcium signaling of both mouse lines may be perturbed in a manner that results in increased membrane excitability. This was illustrated by previous electrophysiological findings that demonstrated abnormal GC excitability in the α-CaMKII hKO mice 15, and similar excitability from the present study in pilocarpine-treated mice.

We further observed increased mRNA expression of calretinin and doublecortin in α-CaMKII hKO mice, but only increased calretinin expression in pilocarpine-treated mice. This finding indicates that there is a similar abnormal fate of newborn neurons in both mouse models. A lack of difference in doublecortin mRNA expression at two months post-administration in the pilocarpine-treated mice compared to vehicle treatment is consistent with previous reports that showed that rates of neurogenesis were high shortly after SE, but gradually declined over the next several months 35, 36. It is likely that at the time the tissue was processed in this study (two months after pilocarpine), doublecortin expression may have already fallen back to near baseline levels in pilocarpine-treated mice. On the other hand, α-CaMKII hKO mice have been shown to constitutively exhibit increased neurogenesis (with bromodeoxyuridine labeling), which is consistent with elevations of doublecortin 15, 19. The difference in doublecortin expression between α-CaMKII hKO and pilocarpine-treated mice suggests that elevations in neurogenesis may not be necessary for the occurrence of an iDG. Thus, in pilocarpine-treated mice, it is likely that the iDG and, perhaps, their behavioral abnormalities occur during the post-SE period of days to weeks during which substantial hippocampal reorganization and neurogenesis occur 42, 43. For example, in the pilocarpine-treated rat, a permanent reduction in hippocampus calbindin mRNA and protein expression was maximal after one month post-SE 26, a period where daily SRS has already developed. Prior to this time-point (especially before one week post-SE), calbindin mRNA and protein expression was nearly indistinguishable from that of controls. In sum, we suspect that there may be an overlap of approximately two to three weeks (occurring at three to six weeks post-SE) during which the iDG is most similar between α-CaMKII hKO and pilocarpine-treated mice. In the present study, we specifically chose the two months post-SE time-point because the seizure mice would be in a chronic ‘epileptic’ state, a condition represented by the majority of human epileptics.

In addition, we have confirmed the abnormal expression of other genes (iDG markers) in α-CaMKII hKO mice from our microarray, namely Tdo2, Bdnf, Drd1a, Grp, Il1r1, and Dsp. Tdo2 and Dsp are maturation marker genes that were highly expressed in the DG, and showed significant down-regulation in both α-CaMKII hKO and pilocarpine-treated mice. The Il1r1 gene, in particular, has been implicated to play a role in the proliferation of neural progenitor cells 44. Several studies have demonstrated that Il1r1 mRNA is highly expressed in the DG GC bodies 45, 46, but their functional role is unclear. It is possible that the DG GCs may contribute to neuronal excitation differently in the two mouse models. The lack of alteration in Il1r1 mRNA expression in pilocarpine-treated mice, and not in α-CaMKII hKO mice, may arise from the fact that SRS may potentiate Il1r1 expression to compensate for excessive excitation in the DG. It has previously been shown that Il1r1 signaling may have anticonvulsive properties 47.

### Dentate gyrus granule cells from pilocarpine-treated mice are physiologically immature

We conducted electrophysiological experiments to evaluate whether the pilocarpine-treated GCs exhibited the iDG phenotype. In agreement with previous studies 48, 49, GCs exhibited less polarized resting membrane potentials and lower spike threshold currents in pilocarpine-treated mice, which resulted in their higher electrophysiological excitability compared to controls. A similar finding was reported in GCs from α-CaMKII hKO mice 15. These results suggest that immature GCs from both models are more hyper-excitable compared to controls. Further, the enhanced dopamine-induced synaptic potentiation recorded in pilocarpine-treated mice was also observed in the fluoxetine-treated mice that exhibited GC dematuration 39. We further applied sustained depolarizing currents to GCs and demonstrated that pilocarpine-treated mice exhibited a significant increase in repetitive spiking. This was in sharp contrast to the α-CaMKII hKO mice, which showed relatively few spikes following the same stimulation protocol 15. Since GCs in early maturational stages hardly generate repetitive spiking during sustained depolarization (16, 50, 51), these findings again suggest that the two mouse lines are in different stages of GC immaturity. DG GCs in α-CaMKII hKO mice resemble newly generated immature neurons, while the GCs from pilocarpine-treated mice resemble neurons that have had a reversal of their maturation, a phenomenon that was observed in chronic fluoxetine-treated mice 16. The intact frequency facilitation at mossy fiber synapses in the pilocarpine-treated mice suggests that their DG output is not in an immature state, but is rather closer to that observed in maturity. Thus, we propose that, similar to what was observed as a result of pilocarpine treatment in mice, seizure-induced changes in the epileptic brain may lead to a ‘dematuration’ of GC neurons resulting in an iDG. Overall, while the electrophysiological properties of pilocarpine-treated GCs share common hyper-excitable characteristics with GCs from the α-CaMKII hKO mice, the mechanisms by which iDG was achieved in the two mouse models may reflect disease-specific differences in neural circuitry and the manner in which the DG relays and processes afferent and efferent connections within the hippocampus.

### Potential contribution of reductions of α-CaMKII to dentate gyrus immaturity in pilocarpine-treated mice

Previous studies have shown a down-regulation of CaMKII activity in epilepsy models 52, 53, suggesting that seizures cause molecular changes in CaMKII signaling. The functional consequences of a reduction in α-CaMKII would be expected to impact the physiology of the hippocampus, in effect, changing the excitability within proximal and distal networks 54, 55. Singleton et al. 52 showed that SE caused inhibition of CaMKII activity from hippocampal homogenates of pilocarpine-treated rats. Because CaMKII positively modulates both excitatory and inhibitory synaptic receptor function, perturbations in CaMKII activity would result in alterations of membrane excitability 52, 56. Singleton et al. 52 further speculated that inhibition of CaMKII activity may decrease GABAergic tone by either a disruption of GABA channel function or an impairment in neurotransmitter synthesis and release. If this is true, down-regulation of CaMKII, and subsequent GABAergic loss in the DG may account for the hyper-excitable nature of GCs observed in both mouse lines.

In pilocarpine-treated mice, SRS is an essential requirement for the development of the dematured DG, since mice from our earlier pilot studies that exhibited very mild SE, with no subsequent SRS, failed to induce a dematured phenotype. Additionally, it was also reported that animals that failed to fully develop SE showed no decrease in CaMKII activity 57. These results suggest a strong connection between SRS, dematured DG and CaMKII. In summary, these observations suggest that α-CaMKII hKO mice and pilocarpine-treated mice may exhibit imbalances in excitatory and inhibitory networks, as a result of an α-CaMKII reduction, which may ultimately contribute to the iDG or dematured DG phenotype and subsequent behavioral abnormalities.

## Conclusions

We found similarities in the cellular/molecular, immunohistochemical, electrophysiological, and behavioral phenotypes of the α-CaMKII hKO and pilocarpine-treated mice. Changes in gene expression point to a decrease in mature neuronal markers and a concomitant increase in immature neuronal markers indicating an iDG phenotype, which may contribute to the pathophysiological and behavioral deficits observed in both psychosis and epilepsy.
